# Luteolin Exerts Cardioprotective Effects through Improving Sarcoplasmic Reticulum Ca^2+^-ATPase Activity in Rats during Ischemia/Reperfusion In Vivo

**DOI:** 10.1155/2015/365854

**Published:** 2015-11-22

**Authors:** Changsheng Nai, Haochen Xuan, Yingying Zhang, Mengxiao Shen, Tongda Xu, Defeng Pan, Congwei Zhang, Yanbin Zhang, Dongye Li

**Affiliations:** ^1^Institute of Cardiovascular Disease, Xuzhou Medical College, Xuzhou, Jiangsu 221002, China; ^2^Department of Cardiology, The Affiliated Hospital of Xuzhou Medical College, Xuzhou, Jiangsu 221002, China

## Abstract

The flavonoid luteolin exists in many types of fruits, vegetables, and medicinal herbs. Our previous studies have demonstrated that luteolin reduced ischemia/reperfusion (I/R) injury in vitro, which was related with sarcoplasmic reticulum Ca^2+^-ATPase (SERCA2a) activity. However, the effects of luteolin on SERCA2a activity during I/R in vivo remain unclear. To investigate whether luteolin exerts cardioprotective effects and to monitor changes in SERCA2a expression and activity levels in vivo during I/R, we created a myocardial I/R rat model by ligating the coronary artery. We demonstrated that luteolin could reduce the myocardial infarct size, lactate dehydrogenase release, and apoptosis during I/R injury in vivo. Furthermore, we found that luteolin inhibited the I/R-induced decrease in SERCA2a activity in vivo. However, neither I/R nor luteolin altered SERCA2a expression levels in myocardiocytes. Moreover, the PI3K/Akt signaling pathway played a vital role in this mechanism. In conclusion, the present study has confirmed for the first time that luteolin yields cardioprotective effects against I/R injury by inhibiting the I/R-induced decrease in SERCA2a activity partially via the PI3K/Akt signaling pathway in vivo, independent of SERCA2a protein level regulation. SERCA2a activity presents a novel biomarker to assess the progress of I/R injury in experimental research and clinical applications.

## 1. Introduction

Coronary artery disease (CAD) is a leading cause of death in adults, aggravated by myocardial ischemia/reperfusion (I/R) injury, which is a complex pathophysiological event in acute myocardial infarction (AMI) that subsequently leads to myocardial systolic and diastolic dysfunction [1–3]. Ca^2+^ overload is a major mechanism of myocardial I/R injury brought about by Ca^2+^ uptake from the cytosol into the sarcoplasmic reticulum (SR), which is mediated by Ca^2+^-ATPase (SERCA2a) [4–6]. More importantly, reduced SERCA2a protein expression and/or activity is known to aggravate Ca^2+^ overload [4, 7, 8]. Therefore, new treatments that target disease mechanisms at the cellular and organ levels are needed to halt and reverse the devastating consequences of this process.

The flavonoid luteolin is a polyphenolic compound recognized as an integral component of the human diet that exists in many types of fruits, vegetables, and medicinal herbs. As reported in many studies, luteolin exerts its protective effects through various mechanisms that convey anti-inflammatory, antioxidative, and antiapoptotic effects [1, 2, 4, 9]. For thousands of years, several flavonoid derivatives have been applied to prevent disease and as therapeutic agents in traditional medicine practiced throughout many Asian countries [10]. The cardiovascular protective effects of luteolin have been widely studied. For example, Hertog et al. [11] reported that routine consumption of flavonoids, such as luteolin, can reduce the incidence of AMI and was negatively correlated with the incidence of CAD.

Our team has long been committed to investigating the effects of luteolin on cardiac protection during I/R and heart failure [1, 2, 9]. We previously demonstrated that luteolin inhibited apoptosis and improved cardiomyocyte contractile function through the PI3K/Akt signaling pathway in myocardiocytes subjected to simulated I/R [1]. We also confirmed that both expression and activity of SERCA2a were reduced during I/R injury in isolated rat hearts [2, 9]. However, the in vivo cardioprotective effects of luteolin and its actions on SERCA2a during I/R injury remain largely unknown. Moreover, Zhang et al. [12] found that SERCA2a activity decreased during I/R injury in vivo, independent of SERCA2a protein level modulation during short-term myocardial I/R injury. However, the reperfusion time used in their model may have been too short to detect relevant changes in protein expression because the longest reperfusion duration in their study was only 120 min, although the half-life of SERCA2a is about 5 days. Therefore, whether a prolonged reperfusion duration will influence SERCA2a protein levels or further decrease SERCA2a activity is unclear. Also, how luteolin exerts its cardioprotective effects through the modulation of SERCA2a in vivo has yet to be elucidated.

The aims of this study were to investigate whether luteolin administration by gavage could exert cardiac protective effects and to identify the mechanisms involved in the modulation of SERCA2a activity during I/R injury in vivo.

## 2. Materials and Methods

### 2.1. Study Approval

The study protocol was approved by the Animal Ethics Committee of Xuzhou Medical College (Xuzhou, Jiangsu Province, China) and all procedures were performed to minimize discomfort to the test animals.

### 2.2. Animals and Reagents

Adult male Sprague-Dawley rats (clean grade, *n* = 117; weight, 200–250 g) were bred at Xuzhou Medical College and randomly divided into four groups: (1) sham group (*n* = 36), (2) I/R group (*n* = 36), (3) luteolin plus I/R (Lut + I/R) group (*n* = 36), and (4) luteolin plus the Akt inhibitor LY294002 plus I/R (Lut + LY + I/R) group (*n* = 9). Luteolin (purity > 98%) was purchased from Xi'an Rongsheng Biotechnology Co., Ltd. (Shanxi, China), and dissolved in 0.5% carboxymethyl cellulose sodium (CMC-Na) [13, 14]. LY294002 and antibodies against Akt, phospho-Akt (Thr-308, Ser-473), caspase-3, and *β*-actin were purchased from Cell Signaling Technology, Inc. (Beverly, MA, USA). Antibodies against SERCA2a, Bcl-2, and Bax were purchased from Santa Cruz Biotechnology, Inc. (Santa Cruz, CA, USA). The Ultramicro-ATPase Assay Kit and a lactate dehydrogenase (LDH) release assay kit were purchased from Nanjing Jiancheng Bioengineering Institute (Nanjing, China).

### 2.3. Surgical Protocol, Group Distribution, and Observation Time of Three Experiment Stages

Luteolin (25 mg/mL; 200 mg/kg body weight dissolved in 0.5% CMC-Na solution) was administered daily to rats in the Lut + I/R and Lut + LY + I/R groups by gavage for 2 weeks before surgery, while the sham and I/R groups received an equal volume of 0.5% CMC-Na solution (geometric conversion according to weight) daily by intragastric administration for the same period. Then, the rats were anesthetized by intraperitoneal injection of 10% sodium pentobarbital (150 mg/kg body weight) and supported using a rodent respirator, while cardiac activity was monitored using the BL-420 Data Acquisition and Analysis System (Chengdu TME Technology Co., Ltd., Chengdu, China). The chest was opened via left thoracotomy through the fifth intercostal space to reveal the beating apex of the heart. After pericardiotomy, a silk suture was placed under the left anterior descending coronary artery (LAD) between the left auricle and pulmonary arterial cone, and the ends of the suture were threaded through a small plastic tube and fixed with an artery clamp to form a reversible occlusion of the LAD [12, 15]. The sham group only underwent thoracotomy and pericardiotomy. The LAD of the Lut + LY + I/R group was ligated for 30 min and then LY294002 (0.3 mg/kg body weight) was injected through the right jugular vein 10 min before reperfusion. Finally, the ischemic portion of the myocardium in the apex of the heart was excised.

The experiment was planned in three stages: the first was to observe whether luteolin could exert cardioprotective effects in vivo during I/R; the second was to investigate whether luteolin exerts protective effects through the regulation of SERCA2a protein expression and activity; and the third aimed to confirm whether luteolin regulated SERCA2a activity by activating the PI3K/Akt signaling pathway. Group distribution, observation time, and the duration of each experimental step are detailed in Table 1.

### 2.4. Measurements of Myocardial Infarct Size and LDH Release

After 1 day of reperfusion, the hearts were quickly excised, frozen at −80°C for 2 h, sliced into 2 mm thick sections across the long axis (beginning from cardiac apex), stained with 1% triphenyltetrazolium chloride (TTC; Sigma-Aldrich Corporation, St. Louis, MO, USA) for 20 min at 37°C, and then incubated in 10% formaldehyde solution for 1 h. The infarct region can be distinguished from the normal region as it is stained gray, while the normal zone is stained brick red. The myocardial infarct size was detected by the ratio of the weight of the infarcted tissue to that of the entire left ventricle. LDH release was determined by drawing arterial blood from the carotid artery after 1 day of reperfusion.

### 2.5. Western Blot Analysis

After reperfusion, the ischemic myocardium in the apex of the heart was excised, homogenized in lysis buffer supplemented with the proteinase inhibitor phenylmethanesulfonyl fluoride, incubated on ice for 30 min, and then centrifuged at 12,000 rpm for 20 min. After the supernatant was collected, its protein concentration was quantified using the modified Bradford assay. Briefly, equal amounts of total protein were separated by sodium dodecyl sulfate polyacrylamide gel electrophoresis on 8%–10% gels and then electrophoretically transferred to nitrocellulose membranes, which were then blocked with 5% bovine serum albumin at room temperature for 3 h. Afterward, the membranes were incubated overnight at 4°C with the following primary antibodies: SERCA2a (dilution, 1 : 5000), Bcl-2, Bax, (dilution, 1 : 2000; Santa Cruz Biotechnology, Inc.), *β*-actin, caspase-3, Akt, and p-Akt (Thr-308, Ser-473; dilution, 1 : 2000; Cell Signaling Technology, Inc.). Next, the membranes were incubated with the corresponding secondary antibodies (dilution, 1 : 2000–1 : 10000; Cell Signaling Technology, Inc.) at room temperature for 2 h. Then, the membranes were treated with Super ECL Plus Detection Reagent (GE Healthcare, Amersham, Little Chalfont, Buckinghamshire, UK) and images were captured on film, which were transferred to a computer. The relative densities of the protein bands were then analyzed using Image J 3.0 software (http://imagej.nih.gov/ij/).

### 2.6. Preparation of the SR from Rat Hearts

SR vesicles were obtained according to the method described by Kodavanti et al. [15]. Briefly, 40 mg of ischemic myocardial tissue was homogenized in 1 mL of lysis buffer [in mM, pH 7.4: 50.0 Na_2_HPO_4_, 10.0 ethylenediaminetetraacetic acid (EDTA), and 25.0 NaF]. The homogenate was centrifuged at 13,000 ×g for 20 min at 4°C. Then, the supernatant was collected, centrifuged for 20 min at 13,000 ×g at 4°C, and centrifuged again at 45,000 ×g for 20 min using an ultracentrifuge (Beckman Coulter, Brea, CA, USA). Pellets of the crude membrane vesicles (SR) were suspended in 1 mL of storage buffer (in mM, pH 7.4: 30.0 histidine, 0.25 sucrose, 10.0 EDTA, and 25.0 NaF) and stored at −80°C until being assayed.

### 2.7. Measurement of SERCA2a Activity

SERCA2a activity was measured using the Ultramicro-ATPase Assay Kit (Nanjing Jiancheng Bioengineering Institute) according to the manufacturer's instructions. The principle of the assay is as follows: ATP can be decomposed into ADP and Pi by ATPase; thus the ATPase activity can be determined by measuring the quantity of Pi. As defined, one unit of ATPase activity is the amount of Pi that is decomposed by ATPase per milligram of tissue protein per hour. The final outcomes are described as *μ*mol Pi/mg protein/h. The SERCA2a protein concentration in the extract was determined using the Bradford assay. In general, the assay is composed of two major steps: an enzymatic reaction and a phosphorus reaction.

### 2.8. Statistical Analysis

Comparisons among all groups were conducted by one-way analysis of variance, followed by the Bonferroni post hoc correction test. All statistical analyses were performed using GraphPad Prism 5 software (GraphPad Software, Inc., La Jolla, CA, USA). All data are expressed as means ± standard deviations (SD) and a probability (*P*) value of < 0.05 was considered statistically significant.

## 3. Results

### 3.1. Mortality and Success Rate of Rat Myocardial I/R Surgery

**Figure 1:**
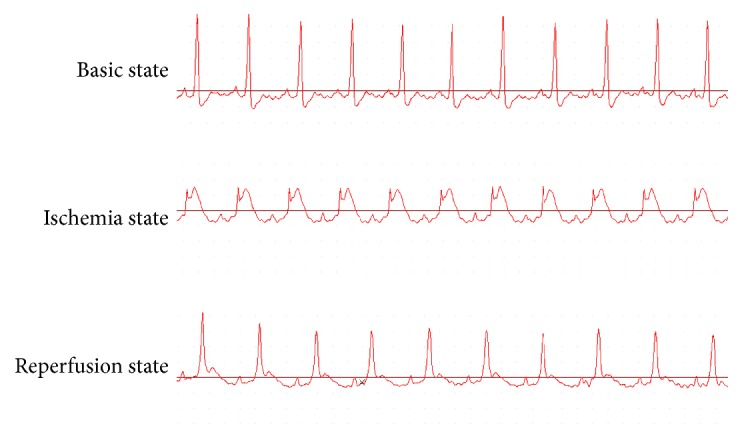
The electrocardiogram (ECG) changes of rats subjected to myocardial I/R injury. Before LAD occlusion, the ST-segment was at baseline. The ST-segment elevated during ischemia duration as compared with basic state, but it declined significantly at reperfusion state.

A total of 125 rats underwent myocardial I/R surgery, of which 117 survived and eight died (two in the sham group, three in the I/R group, two in the Lut + I/R group, and one in the Lut + LY + I/R group), yielding a success rate of 93.6%. During myocardial ischemia and reperfusion, five rats died from severe arrhythmia, two from complete coronary separation injury, and one from ventricular rupture. Changes in electrocardiograms of rats subjected to myocardial I/R injury are shown in Figure 1.

### 3.2. Luteolin Inhibits I/R-Induced Myocardial Infarct Size and LDH Release

**Figure 2:**
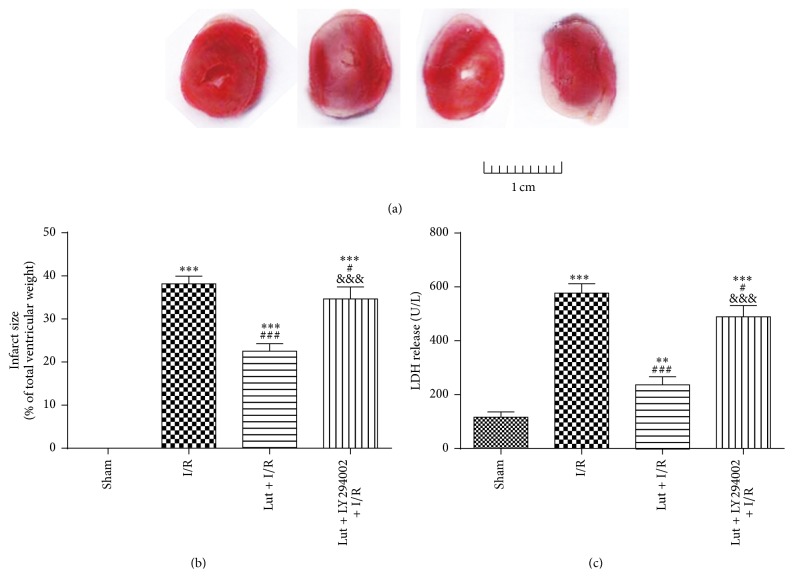
Luteolin inhibits I/R-induced infarct size and LDH release after ischemia for 30 min followed by reperfusion for 1 d. Effects of luteolin and LY294002 on infarct size were shown in (a) and (b). Effects of luteolin and LY294002 on LDH release were shown in (c). The results are expressed as mean ± SD, *n* = 3. ^*∗∗*^ *P* < 0.01, ^*∗∗∗*^ *P* < 0.001 versus sham; ^#^ *P* < 0.05, ^###^ *P* < 0.001 versus I/R; ^&&&^ *P* < 0.001 versus Lut + I/R.

Myocardial infarct size was evaluated by TTC staining and LDH release from ischemic tissues was determined from arterial blood drawn from the carotid after 1 day of reperfusion. As shown in Figure 2, myocardial infarct size and LDH release were increased in the I/R group, as compared with the sham group. The infarct size and LDH release of the Lut + I/R group were decreased, as compared with the I/R group, but both were reversed by pretreatment with LY294002 (Lut + LY + I/R group).

### 3.3. Luteolin Upregulates Antiapoptosis Protein Bcl-2 and Downregulates Proapoptosis Proteins Bax and Cleaved Caspase-3

**Figure 3:**
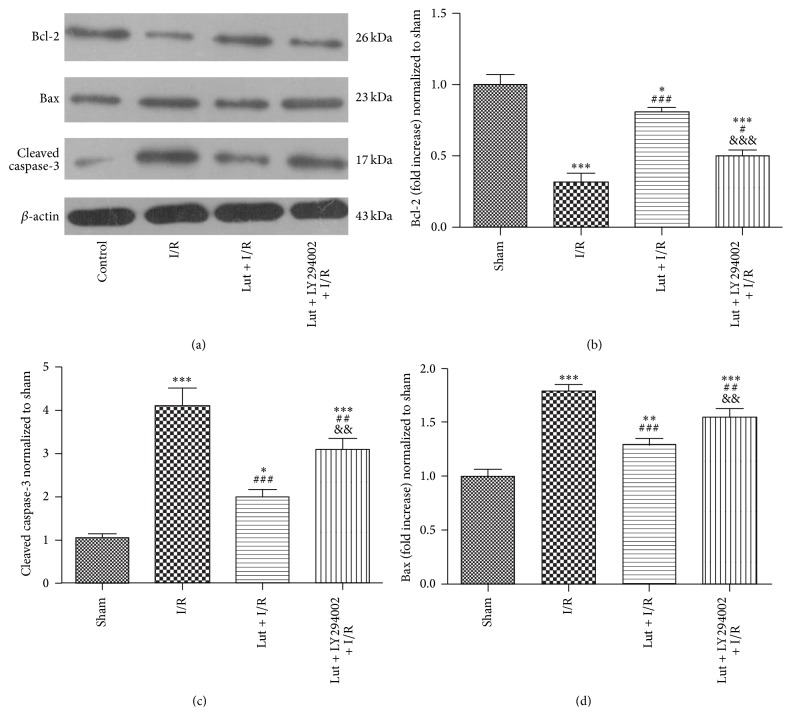
Luteolin upregulates antiapoptosis protein Bcl-2 and downregulates proapoptosis proteins Bax and cleaved caspase-3 after ischemia for 30 min followed by reperfusion for 1 d. The results are expressed as mean ± SD, *n* = 3. ^*∗*^ *P* < 0.05, ^*∗∗*^ *P* < 0.01, and ^*∗∗∗*^ *P* < 0.001 versus sham; ^#^ *P* < 0.05, ^##^ *P* < 0.01, and ^###^ *P* < 0.001 versus I/R; ^&&^ *P* < 0.01, ^&&&^ *P* < 0.001 versus Lut + I/R.

The expression levels of the apoptosis-related proteins Bcl-2, Bax, and cleaved caspase-3 were assessed by western blot analysis after induction of ischemia for 30 min followed by reperfusion for 1 day. As shown in Figure 3, Bax and Bcl-2 expression levels were upregulated and downregulated, respectively, in the I/R group, as compared with the sham group. Bax and Bcl-2 expression levels were downregulated and upregulated, respectively, in the Lut + I/R group, as compared with the I/R group, but were reversed by pretreatment with LY294002 (Lut + LY + I/R group). Cleaved caspase-3 levels were increased in the I/R group, as compared with the sham group, while they were decreased in the Lut + I/R group, as compared with the I/R group, but were reversed by pretreatment with LY294002 (Lut + LY + I/R group).

### 3.4. Luteolin Alleviates the I/R-Induced Decrease in SERCA2a Activity

**Figure 4:**
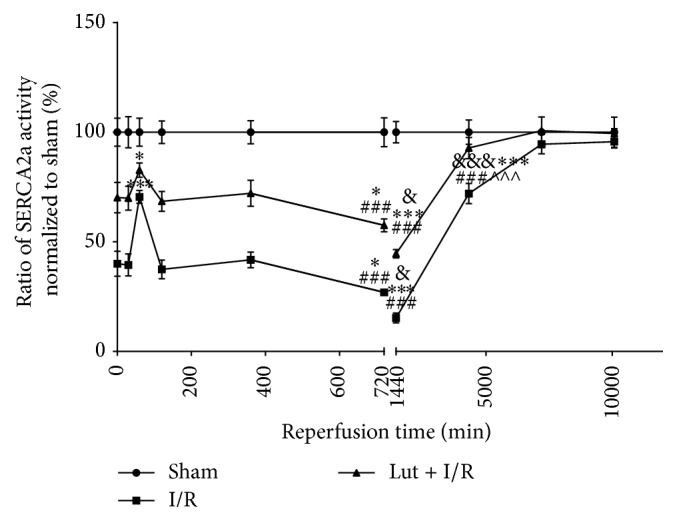
Luteolin alleviates I/R-induced decrease of SERCA2a activity. SERCA2a activities of all sham groups were expressed as 100%, and the other groups were normalized to the corresponding sham groups. This figure was split by different reperfusion duration of 12 h and 1 d. The first half consists of 0 min, 30 min, 60 min, 120 min, 6 h, and 12 h of reperfusion time points. The last half contains 12 h, 1 d, 3 d, 5 d, and 7 d of reperfusion time points. The results are expressed as mean ± SD, *n* = 3. ^*∗*^ *P* < 0.05, ^*∗∗∗*^ *P* < 0.001 versus 30 min/0 min groups; ^###^ *P* < 0.001 versus 30 min/60 min groups; ^&^ *P* < 0.05, ^&&&^ *P* < 0.001 versus 30 min/12 h groups; ^∧∧∧^ *P* < 0.001 versus 30 min/1 d group.

As shown in Figure 4 and Table 2, SERCA2a activities in each sham group over different durations of reperfusion were expressed as 100%, and the I/R and Lut + I/R groups were normalized to the corresponding sham group. Compared with the respective sham groups, SERCA2a activities of all I/R groups in the early reperfusion period (30 min to 3 days) were decreased and were lowest in the I/R group at 30 min and 1 day. However, these activities improved in the Lut + I/R group, as compared with the corresponding I/R groups. During the duration of late reperfusion (3–7 days), SERCA2a activities were totally restored in the 30 min/5-day I/R and 30 min/3-day Lut + I/R groups. There was no difference between the 30/60 min I/R and 60/30 min I/R groups, although the decreases in these two groups were lower than in the 30/0 min, 30/30 min, 30/120 min, and 30 min/6 h I/R groups.

### 3.5. SERCA2a Expression Was Not Influenced by Luteolin Treatment of I/R Injury

**Figure 5:**
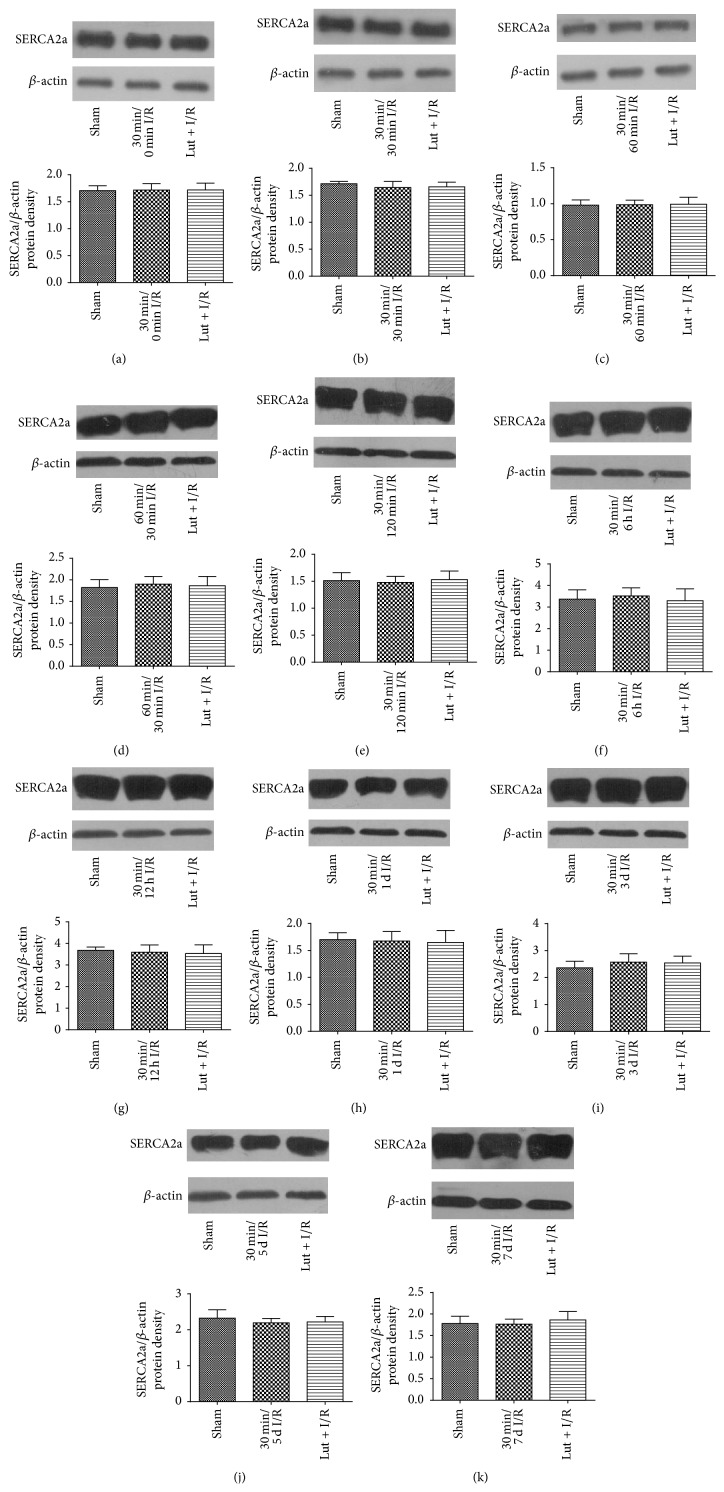
Neither luteolin nor I/R injury changes protein expression of SERCA2a. (a) 30 min/0 min I/R; (b) 30 min/30 min I/R; (c) 30 min/60 min I/R; (d) 60 min/30 min I/R; (e) 30 min/120 min I/R; (f) 30 min/6 h I/R; (g) 30 min/12 h I/R; (h) 30 min/1 d I/R; (i) 30 min/3 d I/R; (j) 30 min/5 d I/R; (k) 30 min/7 d I/R. The results were expressed as ratio of SERCA2a/*β*-actin protein density of 3 independent experiments. There were no significant differences among the studied groups.

As shown in Figure 5, compared with the respective sham group, there was no change in SERCA2a expression level after ischemia for 30 min followed by reperfusion for 0, 30, 60, and 120 min or 6 h, 12 h, 1 day, 3 days, 5 days, and 7 days or ischemia for 60 min followed by reperfusion for 30 min. Moreover, there was no change in SERCA2a expression in the Lut + I/R group.

### 3.6. Luteolin Improves SERCA2a Activity through Partial Activation of the PI3K/Akt Signaling Pathway

**Figure 6:**
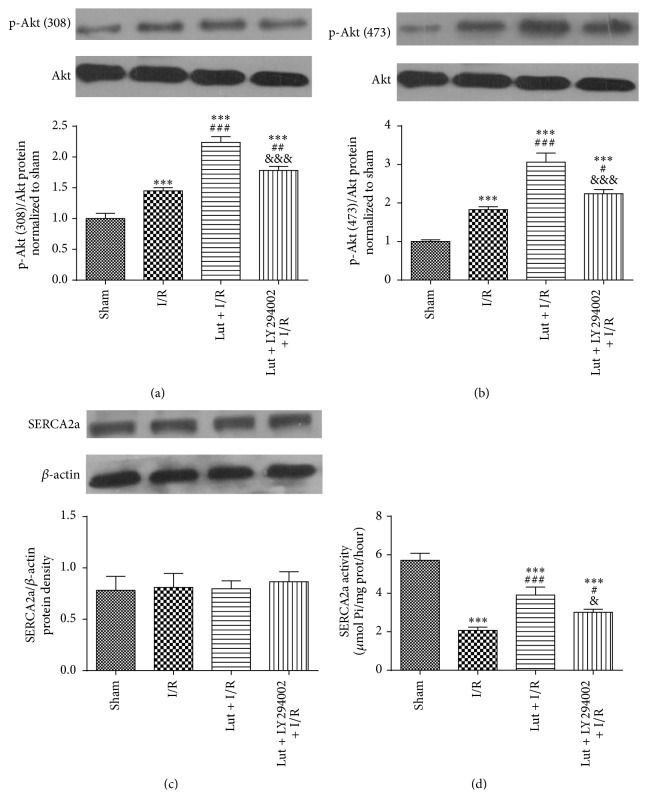
Luteolin improves SERCA2a activity through partially activating PI3K/Akt signaling pathway. The expression levels of p-Akt (308 and 473) and Akt (a and b), SERCA2a protein (c), and SERCA2a activity (d) of four groups which were subjected to ischemia for 30 min followed by reperfusion for 120 min were detected. The results are expressed as mean ± SD, *n* = 3. ^*∗∗∗*^ *P* < 0.001 versus sham; ^#^ *P* < 0.05, ^##^ *P* < 0.01, and ^###^ *P* < 0.001 versus I/R; ^&^ *P* < 0.05, ^&&&^ *P* < 0.001 versus Lut + I/R group.

The expression levels of Akt and phospho-Akt after ischemia for 30 min followed by reperfusion for 120 min were assessed by western blot analysis. As shown in Figures 6(a) and 6(b), as compared with the sham group, levels of p-Akt (Thr-308, Ser-473) were increased 1.45- and 1.83-fold in the I/R group, 2.23- and 3.06-fold in the Lut + I/R group, and 1.78- and 2.24-fold in the Lut + LY + I/R group. However, there were no differences in the protein expression level of total Akt among the four groups. As shown in Figure 6(c), there were no significant differences in SERCA2a expression levels among the four groups (*P* > 0.05).  Figure 6(d) showed that SERCA2a activity was decreased in the I/R group, as compared with the sham group. SERCA2a activity was increased in the Lut + I/R group, as compared with the I/R group, but was reversed by LY294002 pretreatment (Lut + LY + I/R group).

## 4. Discussion

In recent years, the effects of luteolin on I/R injury have become an area of increasing interest. However, most studies have focused on monitoring luteolin levels in vitro using isolated rat hearts or myocardiocytes under certain physiological conditions. Due to the modulation of neuroendocrine factors in vivo, results of these in vitro experiments do not accurately reflect the actual effects of medication [16]. To overcome this limitation, the present study was designed in three experimental stages: first, we investigated whether luteolin exerts cardioprotective effects during I/R in vivo; second, we explored whether luteolin exerts protective effects through regulation of protein expression and activity of SERCA2a; and third we confirmed whether luteolin regulates SERCA2a activity via activation of the PI3K/Akt signaling pathway.

The results of this study primarily confirmed for the first time that luteolin could inhibit I/R-induced myocardial infarct size and LDH release, upregulate antiapoptosis protein Bcl-2, and downregulate proapoptosis proteins Bax and cleaved caspase-3. Furthermore, we confirmed that luteolin exerted cardioprotective effects by improving the I/R-induced decrease of SERCA2a activity in vivo for the first time. Moreover, this study found that increased reperfusion duration led to a further decrease in SERCA2a activity, which was restored by prolonged myocardial I/R duration. Zhang et al. [12] reported that SERCA2a activity decreased after ischemia for 30–60 min followed by reperfusion for 0–120 min. They indicated that SERCA2a activities in the I/R groups observed for 30/0 min, 30/30 min, and 30/120 min all decreased by more than 55%, and the extent of this decrease was similar among these groups (*P* > 0.05). In their opinion, prolonged reperfusion did not lead to a further decrease in SERCA2a activity. In our study, we further investigated changes in SERCA2a activity in response to prolonged reperfusion up to 7 days and found a further decrease in SERCA2a activity, which was lowest in the 30 min/1-day I/R group. However, SERCA2a activity was partially restored in the 30 min/5-day I/R group and totally restored in the 30 min/7-day I/R group. Therefore, we concluded that luteolin could enhance SERCA2a activity, as compared with the I/R groups. Thus, we hypothesized that the reperfusion duration could be divided into two parts: the early (30 min to 3 days) and late (3–7 days) periods. The lowest SERCA2a activity was observed in the 30 min/1-day I/R group. This decrease was likely attributable to the damage caused by I/R injury, which was most severe at a reperfusion time of 1 day [17, 18].

These results suggest that (1) the reperfusion duration could be divided into two parts: early (30 min to 3 days) and late (3–7 days); (2) ischemia for 30 min was sufficient to cause a maximal decline in SERCA2a activity; (3) there was no difference between ischemia/reperfusion for 30/60 min and 60/30 min, although the least decrease was observed in these two groups, as compared with the 30/0 min, 30/30 min, 30/120 min, and 30 min/6 h groups, which can be attributed to the systemic stress response. Hence, this phenomenon may provide guidelines for medication use in the early reperfusion period; (4) in the early reperfusion period (30 min to 3 days), SERCA2a activities of all I/R groups were decreased and were lowest in the 30 min/1-day I/R group, as compared with the sham group, although luteolin improved the decrease in SERCA2a activities, as compared with the corresponding I/R groups; and (5) in the late reperfusion period (3–7 days), the decreases in SERCA2a activity caused by I/R were restored in the 30 min/5-day I/R and 30 min/3-day Lut + I/R groups. The change in SERCA2a activity was similar to those of many clinical biomarkers of AMI (i.e., LDH, CK, CK-MB, cTNI, and cTNT). These results indicate the usefulness of SERCA2a activity as a novel biomarker to assess the progress of I/R injury in experimental research and clinical applications. Further, we deduced that there are some compensatory mechanisms that act to restore SERCA2a activity. As reported in some previous studies, macrophage migration inhibitory factor can be induced at 90 min of I/R and reach maximum activity at 120 min [19, 20], which might partially compensate for the decrease in SERCA2a activity due to its oxidoreductase activity. These results indicated that luteolin exerts cardioprotective effects through amelioration of the I/R-induced decrease in SERCA2a activity in vivo.

We also found that neither luteolin nor I/R injury altered SERCA2a expression when reperfusion was prolonged to 7 days. However, this finding was not consistent with the results reported in other in vitro studies [1, 2]. Although Zhang et al. [12] found no change in SERCA2a expression levels in response to short-term myocardial I/R, the evidence was insufficient to reach a conclusion because the longest reperfusion period in their study was only 120 min, while the half-life of SERCA2a is about 5 days. We confirmed that there were no alterations in SERCA2a expression levels in vivo during I/R because the reperfusion duration of our study was prolonged to 7 days. Of many reports on SERCA2a expression levels in different disease models, Kim et al. [8] demonstrated that SERCA2a expression remained unaltered in wild-type male mice after ischemia for 12 min followed by reperfusion for 1 h using a Langendorff heart perfusion apparatus, while Zhang et al. [21] reported that I/R significantly decreased SERCA2a expression and activity in male SD rats subjected to ischemia for 30 min followed by reperfusion for 2 h using a Langendorff heart perfusion apparatus. These contradictory findings can be explained by the different species, disease models, and especially the obtained in vitro and in vivo levels detailed in these studies.

Physiological processes are complex and mediated by a variety of regulatory mechanisms. Moreover, the exact mechanisms of I/R injury remain unclear. The inconsistent results of these in vitro and in vivo studies indicate that I/R injury influences both organ and neuroendocrine function. Moreover, SERCA2a, which regulates calcium levels in the body, is regulated by a variety of factors, including neuroendocrine activities in I/R injury, which may prevent changes in SERCA2a expression levels. Importantly, these opinions are only hypotheses postulated by the authors, as the exact mechanisms must be further explored in future experiments.

To investigate whether luteolin improved the I/R-induced decrease in SERCA2a activity through activation of the PI3K/Akt signaling pathway, we chose a reperfusion time point of 120 min, which was based on the findings of many studies of the PI3K/Akt signaling pathway [1, 2, 22]. An increasing number of studies have reported that the PI3K/Akt pathway is the most important signaling mechanism involved in the regulation of cell survival [23–26]. Here, we demonstrated for the first time that luteolin improved the I/R-induced decrease in SERCA2a activity in vivo through activation of the PI3K/Akt signaling pathway.

Although Sun et al. [27] pointed out that the protective effects of luteolin were associated with activation of the PI3K/Akt signaling pathway, this finding was not entirely consistent with our results. In their study, the p-Akt/Akt ratio decreased in the I/R group, as compared with the sham group, and luteolin improved the p-Akt/Akt ratio, but there was no significant difference between the luteolin and sham groups. However, in our present study, the p-Akt/Akt ratio was lowest in the sham group but was higher in the I/R group than the sham group and was highest in the Lut + I/R group. Our results are supported by other reports of I/R injury both in vitro [1, 9] and in vivo [24, 28]. Many studies have reported that phospho-Akt levels were not significantly increased in animal models with no acute or chronic damage and the phospho-Akt/Akt ratio was increased in the I/R injury group, as compared with the control group, and significantly upregulated after administration of drugs used in other in vivo studies [24, 28]. Much experimental evidence has indicated that phospho-Akt, which plays a key role in facilitating cell survival, is the active form of Akt [1, 23–26]. In the normal physiological state, phospho-Akt levels are not significantly increased because the Akt protein is not activated. When the body is stimulated (as with I/R injury), protective mechanisms are activated to minimize damage. Under stress conditions, phospho-Akt levels are significantly increased to protect the body. These results, which indicated that luteolin improved the I/R-induced decrease in SERCA2a activity through partial activation of the PI3K/Akt signaling pathway during I/R in vivo, are supported by the findings of many previous studies and definite theories.

Our team has long focused on investigating the protective effects of luteolin on isolated rat hearts and cardiomyocytes during I/R. We had observed that ERK/PP1a/PLB/SERCA2a and JNK pathways were involved in luteolin-mediated protection of rat hearts and cardiomyocytes following I/R [2]. Luteolin inhibited apoptosis and improved cardiomyocyte contractile function through PI3K/Akt pathway in simulated I/R [1]. In the present study, some new findings and novelty are as follows.

The experiment model was different compared with previous studies. The experiment models involved in [1, 2] were in vitro: isolated rat hearts and cardiomyocytes. The isolated rat hearts were subjected to I/R by Langendorff heart perfusion apparatus. Rat cardiomyocytes were achieved by enzyme decomposition and then subjected to simulated I/R by hypoxia/reoxygenation. Strictly speaking, all the studies about luteolin reported above were simulated I/R in vitro. However, in this study, we got a rat heart I/R model by ligation/recanalization of LAD in vivo. Furthermore, the effects of luteolin on rat hearts following I/R injury in vivo were studied.

The results were different compared with previous studies. We previously demonstrated that protein expression of SERCA2a was downregulated after simulated I/R and reversed by luteolin pretreatment through partially activating PI3K/Akt pathway in rat cardiomyocytes [1]. We also had confirmed that both protein expression and activity of SERCA2a were reduced during I/R injury and they could be upregulated by luteolin pretreatment via activation of MAPK (ERK1/2, JNK, and P38) pathway in isolated rat hearts [2]. However, the cardioprotective effects of luteolin and its actions on SERCA2a during I/R in vivo remain unclear.

In this study, firstly, we confirmed for the first time that luteolin could exert cardioprotective effects during I/R in vivo, such as inhibiting I/R-induced myocardial infarct size, LDH release, and apoptosis. Secondly, we found that neither luteolin nor I/R altered SERCA2a protein expression when reperfusion duration was prolonged to 7 days in vivo. However, this finding was not consistent with the results reported in other in vitro studies [1, 2]. Thirdly, we observed that luteolin exerted cardioprotective effects by improving the I/R-induced decrease of SERCA2a activity in vivo for the first time. Fourthly, reperfusion duration was prolonged to 7 days and many reperfusion time points were designed in this study for the first time. We confirmed that SERCA2a activity was the lowest when reperfusion duration was prolonged to 1 day. However, SERCA2a activity was partially restored in the 30 min/5-day I/R status and totally restored in the 30 min/7-day I/R duration. The change in SERCA2a activity was similar to those of many clinical biomarkers of AMI (i.e., LDH, CK, CK-MB, cTNI, and cTNT). These results indicated the usefulness of SERCA2a activity as a novel biomarker to assess the progress of I/R injury in experimental research and clinical applications. Fifthly, we demonstrated that luteolin improved the I/R-induced decrease of SERCA2a activity through partial activation of the PI3K/Akt signaling pathway during I/R in vivo, and this was not completely consistent with results of [2]. In that study, luteolin upregulated SERCA2a activity via activation of MAPK pathway. However, this study confirmed that luteolin improved SERCA2a activity through partially activating the PI3K/Akt pathway. And other signaling pathways just like MAPK pathway might be also involved in this process and this hypothesis needs to be studied deeply in the future.

## 5. Conclusion

In conclusion, the results of the present study indicated for the first time that intragastric administration of luteolin decreased I/R-induced myocardial infarct size, LDH release, and apoptosis of myocardiocytes in rat hearts in vivo. Furthermore, we demonstrated that luteolin exerts cardioprotective effects in rat hearts in vivo through preserving the I/R-induced decrease in SERCA2a activity, partially via the PI3K/Akt signaling pathway, independent of SERCA2a protein level modulation. Due to the tendency for altered activity during I/R injury, SERCA2a presents a novel biomarker to assess the progress of I/R injury in both experimental research and clinical applications.

## Figures and Tables

**Table 1:** Surgical protocol, group distribution, and observation time of three experiment stages.

Experiment stage	Groups	Observation time (*n* = 3 at each time point in each group)	Test items
First step	Sham I/R Lut + I/R Lut + LY + I/R	Ischemia for 30 min followed by reperfusion for 1 d	Myocardial infarct size LDH release Apoptosis-related proteins

Second step	Sham I/R Lut + I/R	Ischemia for 30 min followed by reperfusion for 0, 30, 60, and 120 min; 6, 12 h; 1, 3, 5, and 7 d and ischemia for 60 min followed by reperfusion for 30 min	Protein expression and activity of SERCA2a

Third step	Sham I/R Lut + I/R Lut + LY + I/R	Ischemia for 30 min followed by reperfusion for 120 min	Protein expressions of Akt, p-Akt 308, and p-Akt 473 Protein expression and activity of SERCA2a

**Table 2:** Ratio of SERCA2a activity of three groups at each reperfusion time point.

Group	30 min/0 min I/R	30 min/30 min I/R	30 min/60 min I/R	60 min/30 min I/R
Sham	100%	100%	100%	100%
I/R	(40.06 ± 4.64)%^*∗*^	(39.46 ± 4.10)%^*∗*^	(70.52 ± 2.40)%^*∗*&^	(70.89 ± 1.66)%^*∗*&^
Lut + I/R	(70.18 ± 5.63)%^*∗*#^	(69.92 ± 4.42)%^*∗*#^	(82.72 ± 2.66)%^*∗*#&^	(86.25 ± 1.82)%^*∗*#&^

Group	30 min/120 min I/R	30 min/6 h I/R	30 min/12 h I/R	30 min/1 d I/R

Sham	100%	100%	100%	100%
I/R	(37.44 ± 3.48)%^*∗*^	(41.80 ± 2.93)%^*∗*^	(26.99 ± 1.16)%^*∗*&$^	(15.28 ± 1.84)%^*∗*&$*¥*^
Lut + I/R	(68.46 ± 3.69)%^*∗*#^	(72.15 ± 4.83)%^*∗*#^	(57.51 ± 2.40)%^*∗*#&$^	(44.57 ± 1.57)%^*∗*#&$*¥*^

Group	30 min/3 d I/R	30 min/5 d I/R	30 min/7 d I/R	

Sham	100%	100%	100%	
I/R	(72.04 ± 3.73)%^*∗*&*¥*@^	(94.53 ± 3.58)%^&*¥*@∧^	(95.69 ± 2.32)%^&*¥*@∧^	

The number of groups is too much to describe in detail, so *P* < 0.05 was considered statistically significant uniformly.

^*∗*^ *P* < 0.05 versus sham group. ^#^ *P* < 0.05 versus I/R group. ^&^ *P* < 0.05 versus 30 min/0 min group

^$^ *P* < 0.05 versus 30 min/60 min group. ^*¥*^ *P* < 0.05 versus 30 min/12 h group.

^@^ *P* < 0.05 versus 30 min/1 d group. ^∧^ *P* < 0.05 versus 30 min/3 d group.
